# The combined risk effect among *BIN1*, *CLU,* and *APOE* genes in Alzheimer’s disease

**DOI:** 10.1590/1678-4685-GMB-2018-0320

**Published:** 2020-03-16

**Authors:** Lígia Ramos dos Santos, Jucimara Ferreira Figueiredo Almeida, Lúcia Helena Sagrillo Pimassoni, Renato Lírio Morelato, Flavia de Paula

**Affiliations:** 1 Universidade Federal do Espírito Santo, Centro de Ciências Humanas e Naturais, Departamento de Ciências Biológicas, Núcleo de Genética Humana e Molecular, Vitória, ES, Brazil.; 2 Universidade Federal do Espírito Santo, Programa de Pós-Graduação em Biotecnologia, Vitória, ES, Brazil.; 3 Escola Superior de Ciências da Santa Casa de Misericórdia de Vitória, Vitória, ES, Brazil.; 4 Hospital da Santa Casa de Misericórdia de Vitória, Escola Superior de Ciências da Santa Casa de Misericórdia de Vitória, Vitória, ES, Brazil.

**Keywords:** GWAS variants, APOE, CLU, BIN1, ABCA7

## Abstract

Genome-wide associations studies (GWAS) are detecting new variants associated with late-onset of Alzheimer’s disease (LOAD), a multifactorial neurodegenerative disorder. The variants rs744373 *BIN1,* rs11136000 *CLU* and rs3764650 *ABCA7* uncovered by GWAS led to different AD pathways, such as metabolism, trafficking and endocytosis of lipids and inflammation. However, most of the association studies did not replicate these variants with significance. This could be due to a small power effect evident when these variants are tested independently with LOAD. Therefore, we aimed to investigate whether the combination of different variants would additively modify the risk of association with LOAD that is observed in GWAS. We performed an association study testing pairwise variants in metabolism, trafficking and endocytosis of lipid (rs429358 and rs7412 *APOE*, rs744373 *BIN1*, rs3764650 *ABCA7* and rs11136000 *CLU)* pathways with LOAD in samples from southeastern Brazil. Our data suggest a risk effect for LOAD between *APOE* with *CLU* and *APOE* with *BIN1* genes.

## Introduction

Alzheimer’s disease (AD) is a neurodegenerative disease that affects millions of elders globally (Prince *et al.*, 2016). Familial, or early-onset AD (EOAD), accounts for 2% of AD cases and occurs before 65 years. EOAD has Mendelian patterns of inheritance, with mutations in *APP* (amyloid precursor protein), *PSEN1* (presenilin 1) and *PSEN2* (presenilin 2) genes (Bertram *et al.*, 2010; Holtzman *et al.*, 2011). Unlike EOAD, late-onset AD (LOAD) has a multifactorial pattern, with influence of genetic and environmental factors. It occurs after 65 years and accounts for 98% of AD cases (Yu *et al.*, 2014). To date, the ε4 allele in the apolipoprotein E (*APOE*) gene is considered a major risk factor for LOAD worldwide (Lambert and Amouyel, 2011).

The main hypothesis regarding neurodegeneration in AD is that the amyloid cascade leads to amyloid plaque formation (Heppner *et al.*, 2015). This event occurs due to the impaired degradation of neurotoxic Aβ42 peptides. Both the increased formation and the decrease in the clearance of Aβ42 peptides, is considered to play a role in the development of AD. Recent studies suggest that cholesterol is a part of the regulation in the clearance of Aβ42 peptides formed in the brain (Kojro *et al.*, 2001; O’Brien *et al.*, 2011; Reitz, 2013). In neurons, cholesterol is vital for function and plasticity. Function and plasticity are important in the process of learning and memory formation, all of which are found to be impaired in AD (Pfrieger, 2003). Moreover, several genes beyond APOE have been implicated in alterations in cholesterol metabolism, trafficking and endocytosis, such as Clusterin *(CLU),* Bridging integrator 1 *(BIN1)* and the ATP-binding cassette transporter A7 (*ABCA7*) genes, all variants that have been identified in genome-wide associations studies (GWAS) (Harold *et al.*, 2009; Lambert *et al.*, 2009; Hollingworth *et al.*, 2011; Naj *et al.*, 2011; Karch and Goate, 2014). Most of the GWAS variants associated with LOAD have small effects individually (Ebbert *et al.*, 2015). In addition, the case-control studies that replicated those variants did not all reach significance. In this scenario, the nonsignificance may be due to a lack of the power effect of those variants when tested independently with LOAD. It is possible that a combination of different variants together would enhance the effect of association with LOAD that is observed in GWAS. Therefore, the main goal of this study was to test pairwise variants from metabolism, trafficking and endocytosis of lipid (rs429358 and rs7412 *APOE*, rs744373 *BIN1*, rs3764650 *ABCA7* and rs11136000 *CLU)* pathways with late-onset AD in a sample from southeastern Brazil.

## Subjects and Methods

### Subjects

This is an association study with 224 unrelated individuals. We selected 79 elderly patients diagnosed for probable AD with LOAD according to the criteria of the National Institute of Neurological and Communicative Disorders and Stroke and the Alzheimer disease and Related Disorders Association (NINCDS-ADRDA). These patients had a comprehensive diagnostic evaluation for dementia and fulfill other criteria, such as the Mini-Mental State Examination (MMSE). For controls, 145 healthy elderly patients were selected, and all were matched for sex and age. Demographic and clinical data of the sample composition is presented in Table 1. Our recent study (dos Santos *et al.*, 2017) demonstrated that our sample had no difference for variables, such as gender (*p*=0.536), ethnic background (*p*=0.641), schooling (*p*=0.281) and age (*p*=0.144), except for APOE status (*p* < 0.001), among AD cases and controls.

**Table 1:** Sample characteristics.

Variable	Controls 145 (100%)	AD Patients 79 (100%)
Gender		
Woman	106 (73.1%)	54 (68.4%)
Man	39 (26.9%)	25 (31.6%)
Ethnic background		
Caucasians	83 (57.2%)	45 (57.0%)
Afro-Brazilians	60 (41.4%)	34 (43.0%)
No identification	2 (1.4%)	0
Schooling		
Literate	94 (64.8%)	41 (51.9%)
Illiterate	45 (31.0%)	28 (35.4%)
No identification	6 (4.1%)	10 (12.7%)
APOE status		
ε4 -	102 (70.3%)	35 (44.3%)
ε4 +	43 (29.7%)	44 (55.7%)
Age (mean and SD)	80,1 ± 7,8	81,6 ± 7
MMSE	> 28	14-4

AD Patients = Alzheimer’s disease patients; ε4 += ε4 carriers; ε4 -= ε4 non-carriers; SD= standard deviation; Mini-Mental State Examination (MMSE).

All the participants in this research resided in the metropolitan region of Espírito Santo, Grande Vitória, in southeastern Brazil. They were assisted by a geriatrician at the Geriatric Unit of the Hospital Santa Casa de Misericórdia de Vitória (HSCMV) and Centro de Atendimento ao Idoso (CRAI), ES, Brazil. Additionally, the participants or their relatives gave written informed consent agreeing to participate in the research study. Information regarding age, gender, ethnic background composition and schooling was collected. The geriatrician meticulously selected only participants with no family history of Alzheimer’s. This study was accepted by the Committee of Ethics in Human Research of Escola Superior de Ciências da Santa Casa de Misericórdia de Vitória, Brazil.

### Blood sampling and genotyping

Peripheral blood was collected into a 5 mL tube with 5% ethylene diamine tetraacetic acid (EDTA) at the Geriatric Unit of HSCMV and CRAI. The samples were stored at 4 °C prior to analyses. Genomic DNA was isolated according to previous methodology (Miller *et al.*, 1988). We calculated the adequate sample size using the proportion of the genes in the population based on the overall frequency of the minor alleles of each polymorphism. The estimate of adequate sample size for the *APOE* gene and *ABCA7* polymorphisms was 207, and for the *CLU* and *BIN1* genes it was 377 individuals. Therefore, our results are consistent since our sample contained 224 individuals.

Genotyping was performed by our previous coworkers (Almada *et al.*, 2012; Belcavello *et al.*, 2015; dos Santos *et al.*, 2016, 2017) using the Brazilian sample set of this study. The variant rs3764650 *ABCA7* was performed by Santos *et al.* (2017) through real time - polymerase chain reaction (qPCR), and the three standard genotypes were confirmed by Sanger sequencing. Analysis of the variants rs744373 *BIN1,* rs11136000 *CLU* and (rs429358 and rs7412) *APOE* were performed, respectively, by Almada *et al.* (2012), Belcavello *et al.* (2015) and dos Santos *et al.* (2016) thought restriction fragment length polymorphism - polymerase chain reaction (RFLP-PCR).

### Statistical analysis

All the statistical analyses were performed using SPSS (IBM) software v.23.0 for Windows. A *p*-value < 0.05 was considered significant.

Logistic regression analysis was performed for each single nucleotide polymorphism (SNP) and for allelic combinations between two polymorphisms. The *p*-value was adjusted using *APOE* status, age, gender, school level and ethnic background as variables. For education, was considered literate or illiterate. For *APOE* status, was considered an ε4 + for those that carried at least one ε4 allele; and ε4 -, for those that carried no ε4 allele. The *p*-value in *APOE* association with LOAD when pairwise with another variant, or not combined, was not adjusted for *APOE* status. The allelic combinations tested followed genes from lipid metabolism and the endocytosis pathway (*ABCA7, CLU, BIN1* and *APOE*). The allele frequencies of SNPs were inferred from the following studies: dos Santos *et al.* (2017) for rs3764650 *ABCA7;* dos Santos *et al.* (2016) for rs744373 *BIN1*, Belcavello *et al.* (2015) for rs11136000 *CLU,* and Almada *et al.* (2012) for rs429358 and rs7412 *APOE.*

## Results

Results of the test of independent association for LOAD of *CLU* (rs11136000), *ABCA7* (rs3764650), *BIN1* (rs744373) and *APOE* (rs429358 and rs7412) are presented in Table 2. As expected, the ε4 allele in *APOE* was statistically significant. No association was observed for LOAD for the G allele in *ABCA7*, the T allele in *CLU* and the C allele in *BIN1* genes.

**Table 2:** Test for independent interaction of SNPs with LOAD.

Polymorphism	AD Patients N (%)	Controls N (%)	OR (95% IC)	*p*-value^a^	OR (95% IC)	*p*-value^b^
*ABCA7* (rs3764650)						
T	52 (68.4%)	105 (75.5%)	1 (Reference)	-	1 (Reference)	-
G	24 (31.6%)	34 (24.5%)	1.425 (0.767-2.648)	0.262	1.552 (0.794-3.037)	0.199
*CLU* (rs11136000)						
C	89 (57.1%)	170 (58.6%)	1 (Reference)	-	1 (Reference)	-
T	67 (42.9%)	120 (41.4%)	0.938 (0.632-1.390)	0.764	0.840 (0.537-1.314)	0.445
*BIN1* (rs744373)						
T	106 (67.1%)	186 (65%)	1 (Reference)	-	1 (Reference)	-
C	52 (32.9%)	100 (35%)	0.912 (0.605-1.377)	0.662	0.960 (0.606-1.520)	0.861
*APOE* (rs429358, rs7412)						
ε4 -	103 (65.2%)	245 (84.5%)	1 (Reference)	-	1 (Reference)	-
ε4 +	55 (34.8%)	45 (15.5%)	2.907 (1.842-4.588)	< 0.001	3.029 (1.873-4.898)	< 0.001 ^c^

ε4 - = ε4 non-carriers; ε4+ = ε4 carriers; AD Patients = Alzheimer’s disease patients; OR = odds ratio; CI = confidential interval;*p-*value considerer ≤ 0.05; ^a^ = crude *p*-value; ^b^ = *p*-value adjusted by the variables age, gender, educational attainment, ethnic background and APOE ε4 status; ^c^ = *p*-value adjusted by the variables on ^b^ except for APOE ε4 status.

The data of combined allelic variants are presented in Table 3. A significant association was not observed between *CLU* and *ABCA7, CLU* and *BIN1* or *ABCA7* and *BIN1*. The presence of the ε4 allele in *APOE* alone was associated with LOAD in the absence of the minor G allele *ABCA7* (*p* < 0.001)*,* the absence of the minor C allele in *BIN1* (*p* < 0.001) and the T allele in *CLU* (*p*=0.030), also after p-value adjustment. The presence of the C allele in *BIN1* and the ε4 allele in *APOE* showed risk for LOAD (OR = 3.489), even after *p*-value adjustment (OR = 3.678). The presence of both T alleles in *CLU* and the ε4 allele in *APOE* enhances the risk 3.911-fold for LOAD and after *p*-value adjustment (OR = 3.633). However, no association was found between the ε4 allele in *APOE* and the G allele in *ABCA7* (*p*= 0.128) and after *p*-value adjustment (*p*=0.115).

**Table 3:** Combined allelic effect among variants in the study.

Genes		AD Patients N (%)	Controls N (%)	OR (95% IC)	*p-*value^a^	OR (95% IC)	*p*-value^b^
*ABCA7* (G)	*APOE* (ε4)						
-	-	88 (57.1%)	213(73.9%)	1 (Reference)	-	1 (Reference)	-
-	+	40 (26%)	31 (10.8%)	3.123 (1.837-5.310)	<0.001	3.459 (1.977-6.052)	< 0.001
+	-	15 (9.8%)	30 (10.4%)	1.210 (0.621-2.360)	0.575	1.391 (0.702-2.755)	0.344
+	+	11 (7.1%)	14 (4.9%)	1.902 (0.831-4.352)	0.128	2.014 (0.844-4.806)	0.115
*BIN1* (C)	*APOE* (ε4)						
-	-	61 (38.6%)	149(52.1%)	1 (Reference)	-	1 (Reference)	-
-	+	45 (28.5%)	37 (13.0%)	2.971 (1.753-5.033)	<0.001	3.376 (1.926-5.918)	< 0.001
+	-	42 (26.6%)	93 (32.5%)	1.103 (0.689-1.766)	0.683	1.313 (0.788-2.187)	0.297
+	+	10 (6.3%)	7 (2.4%)	3.489 (1.270-9.588)	0.015	3.678 (1.275-10.616)	0.016
*CLU* (T)	*APOE* (ε4)						
-	-	57 (36.5%)	138(47.6%)	1 (Reference)	-	1 (Reference)	-
-	+	32 (20.5%)	32 (11%)	2.421 (1.357-4.320)	0.030	2.664 (1.450-4.893)	0.020
+	-	46 (29.5%)	107(36.9%)	1.041 (0.655-1.654)	0.860	1.055 (0.640-1.741)	0.834
+	+	21 (13.5%)	13 (4.5%)	3.911 (1.834-8.341)	< 0.001	3.633 (1.628-8.107)	0.020
*BIN1* (C)	*ABCA7* (G)						
-	-	79 (51.3)	148 (52.1)	1 (Reference)	-	1 (Reference)	-
-	+	24 (15.6)	36 (12.7)	1.249 (0.696-2.240)	0.456	1.474 (0.786-2.770)	0.226
+	-	49 (31.8)	94 (33.1)	0.977 (0.629-1.517)	0.916	1.077 (0.658-1.763)	0.768
+	+	2 (1.3)	6 (2.1)	0.624 (0.123-3.166)	0.570	0.824 (0.148-4.586)	0.825
*BIN1* (C)	*CLU* (T)						
-	-	66 (42.3)	129 (45.1)	1 (Reference)	-	1 (Reference)	-
-	+	38 (24.4)	57 (19.9)	1.303 (0.785-2.162)	0.306	1.002 (0.565-1.775)	0.995
+	-	23 (14.7)	38 (13.3)	1.183 (0.651-2.149)	0.581	1.336 (0.691-2.584)	0.389
+	+	29 (18.6)	62 (21.7)	0.914 (0.517-1.555)	0.741	0.786 (0.431-1.435)	0.433
*ABCA7* (G)	*CLU* (T)						
-	-	67 (43.5)	131 (45.5)	1 (Reference)	-	1 (Reference)	-
+	-	22 (14.3)	37 (12.9)	1.163 (0.635-2.127)	0.625	1.375 (0.715-2.641)	0.340
-	+	61 (39.6)	113 (39.2)	1.055 (0.688-1.620)	0.805	0.914 (0.562-1.486)	0.717
+	+	4 (2.6)	7 (2.4)	1.117 (0.316-3.952)	0.863	0.719 (0.190-2.719)	0.627

+ / - = allelic presence / allelic absence; AD Patients = Alzheimer’s disease patients; OR = odds ratio; CI = confidential interval; p-value considerer ≤ 0.05; ^a^ = crude *p-*value; ^b^ = *p*-value adjusted by the variables age, gender, educational attainment and ethnic background.

## Discussion

LOAD studies of additive combinations of genetic variants are scarce, and most of those published articles had non-GWAS variants. In this study, we aimed to investigate GWAS variants combined and with the *APOE* gene as well, for late-onset AD in samples from southeastern Brazil. Among the combination tested, we found a risk for LOAD between *CLU* and *APOE* and between *BIN1* and *APOE* genes.

The Apolipoprotein E (*APOE*) gene is localized at chromosome region 19q13.2 and encodes the APOE protein, an apolipoprotein (Morgan and Carrasquillo, 2013). Due to the SNPs rs429358 and rs7412 in the *APOE* gene, three haplotypes are formed: ε2 (T allele rs7412 and T allele rs429358), ε3 (C allele rs7412 and T allele rs429358), and the ε4 allele (C allele rs7412 and C allele rs429358) (Morgan and Carrasquillo, 2013). The APOE protein and Clusterin (CLU) protein, another apolipoprotein, carries cholesterol among brain cells (El Gaamouch *et al.*, 2016) and acts on the clearance of Aβ peptides (Rizzi *et al.*, 2009). The CLU or Apolipoprotein J (APOJ) protein (Rizzi *et al.*, 2009) is associated with a neuroprotective effect in AD (Schrijvers *et al.*, 2011). APOJ is encoded by the *Clusterin* (*CLU*) gene, located at chromosome 8p21.1 (Schrijvers *et al.*, 2011). The *CLU* gene has the T allele from the polymorphism rs11136000 as a protective factor associated with LOAD in the later GWAS (Harold *et al.*, 2009; Lambert *et al.*, 2009). We found that the T allele in the rs11136000 *CLU* gene is not related to LOAD independently. Our result is corroborated with the new association studies (Tan *et al.*, 2016; Shankarappa *et al.*, 2017) of rs11136000 that did not find an association for LOAD in a population of 407 individuals from India (Shankarappa *et al.*, 2017), and 329 individuals from United States (Tan *et al.*, 2016). In combined variant tests, we found that the T allele in rs11136000 *CLU* in combination with the ε4 allele in *APOE,* enhances the odds of risk for LOAD. A possible explanation is that the rs11136000 variant may be underpowered alone in our sample and when in a combination with the ε4 allele in APOE. This variant may modulate the protectiveness aspect in carriers of the T allele in order to favor risk for AD. We believe that both genes may have functional implications in AD pathology. For instance, studies have shown that the lipidated APOE and CLU proteins can bind to Aβ peptides individually to direct them to clearance in the brain (Tokuda *et al.*, 2000). Additionally, another study demonstrated that in PDAPP transgenic mice, the absence of APOE and CLU proteins affects the clearance of Aβ peptides (DeMattos *et al.*, 2004). This suggests that *APOE* and *CLU* genes may regulate this function together.

The *Bridging integrator 1 (BIN1)* gene is located at chromosome 2q14.3 and has the SNP rs744373 associated as a risk factor to LOAD (Harold *et al.*, 2009). The *BIN1* gene encodes the BIN1 protein, which is related to intracellular endosome trafficking of lipids and clathrin mediated endocytosis (Pant *et al.*, 2009). In AD, BIN1 may impact trafficking and endocytosis of cholesterol in the brain and the clearance of Aβ peptides, since it may not internalize with efficiency (Pant *et al.*, 2009; Dong *et al.*, 2017). In our data, the C allele in *BIN1* is not independently associated with LOAD. This result is also observed in the work of Hohman *et al.* (2013) in a population from United States (n=235) and in Li *et al.* (2015) in a study of the Han Chinese population (n=554). In our work, the combination of the C allele of rs744373 *BIN1* and the ε4 allele of APOE is risk association (*p*=0.015) for LOAD. We believe that *BIN1* and *APOE* may have a possible relation in AD. The study of Lazaris *et al.* (2015), for example, reported that the CC genotype in rs744373 *BIN1* modulates the association between plasma levels of APOE and brain amyloidosis, which implies evidence of the interaction between the *BIN1* and *APOE* genes.

The SNP rs3764650 in the *ABCA7* gene was reported by GWAS to be a risk factor for LOAD (Hollingworth *et al.*, 2011). The *ATP-binding cassette transporter A7* (*ABCA7*) gene is a member of the ABC transporters and is located at chromosome 19p13.3. This gene encodes the ABCA7 protein, which actively translocates lipids, such as cholesterol, through cell membranes to APOE and lipidated APOE (Abe-Dohmae *et al.*, 2004; Vasiliou *et al.*, 2009). In the present study, the G allele in rs3764650 *ABCA7* is not associated with LOAD, neither separately nor in combination with the ε4 allele in *APOE* genes. A study by Yamazaki *et al.* (2017) of 100 Japanese patients, and a study by Hohman *et al.* (2013) consisting of 238 American patients also did not find independent association with LOAD. Although our data found no relation of *APOE* with the *ABCA7* gene, studies support a role for ABCA7 in lipidation of the APOE protein with cholesterol and Aβ peptide clearance in the pathogenesis of AD (Kim *et al.*, 2008). For instance, an *in vitro* study by Chan *et al.* (2008) reported that *ABCA7* stimulates cholesterol efflux to *APOE*, and can suppresses Aβ production.

Our work found that the gene combinations of *CLU* with *APOE* and *BIN1* with *APOE* additively modify the risk of association with LOAD. However, we cannot ignore the possibility of a false positive result. This is due to the overpowering effect of ε4 *APOE* alone, disregarding the gene combination. Nevertheless, the possibility of a false-positive result is little plausible, since we did not find an association of the ε4 allele in *APOE* with the G allele in *ABCA7*. Regarding the data of combined variants that had no association for LOAD in our data, the Brazilian population is a combination of Iberian Caucasians, West Africans, and Native Americans (Lins *et al.*, 2010; Pena *et al.*, 2011). Such ethnic profiles might be responsible for different allele frequencies that may favor risk factors in each population. Moreover, late-onset AD is a complex disease with diverse components in its interactions, such as epigenetics, age, environment, sex, and genetics (Combarros *et al.*, 2009). With respect to genetic factors, no single polymorphism can fully explain the disease (Dong *et al.*, 2017). Rather, it is a combination of gene variants that may enlighten the concepts of susceptibility to AD (Vepsäläinen *et al.*, 2009). We believe our results are important to enhance the understanding in the underling etiology of the disease and establishment of novel therapeutic approaches for AD.

## Conclusion

Our data suggest that combinations of variants in *CLU* with *APOE* and *BIN1* with *APOE* genes are associated with LOAD in the southeast Brazilian population.
